# Moderating effect of APOE ε4 on the association of sleep disturbance and amyloid-β pathology among cognitively normal older adults

**DOI:** 10.3389/fnagi.2025.1627774

**Published:** 2025-09-17

**Authors:** Shufei Feng, Jianyu Que, Qianwen Wang, Kai Yuan, Le Shi

**Affiliations:** ^1^Peking University Sixth Hospital, Peking University Institute of Mental Health, NHC Key Laboratory of Mental Health (Peking University), National Clinical Research Center for Mental Disorders (Peking University Sixth Hospital), Beijing, China; ^2^Xiamen Xianyue Hospital, Xianyue Hospital Affiliated with Xiamen Medical College, Fujian Psychiatric Center, Fujian Clinical Research Center for Mental Disorders, Fujian, China

**Keywords:** Alzheimer’s disease, amyloid-β, APOE ε4, moderating analysis, sleep disturbance

## Abstract

**Background:**

Sleep–wake rhythms are critical for the development of Alzheimer’s disease (AD). However, the relationship of sleep disturbance, APOE ε4, and amyloid-β (Aβ) accumulation remains unclear. Thus, this study investigated the potential role of APOE ε4 allele in the association between sleep disturbance and brain Aβ burden among cognitively normal (CN) older adults.

**Methods:**

In this cross-sectional study, data were obtained from the Alzheimer’s Disease Neuroimaging Initiative (ADNl) Database. The sample consisted of CN individuals aged between 55 and 90 years with Aβ positron emission tomography scan, APOE genotype, and sleep assessment using the Neuropsychiatric Inventory.

**Results:**

The study included 1,000 CN participants, including 134 individuals with sleep disturbances and 306 APOE ε4 carriers (APOE ε4+). After adjusting for sex, age, years of education, and marital status, sleep disturbance was not associated with a higher Aβ burden among participants. However, a significant interaction between sleep disturbance and APOE ε4 on regional standardized uptake value ratios was observed, such as in the left hippocampus. Subgroup analysis revealed that sleep disturbance could affect the AD-sensitive brain regions in the APOE ε4 + group. Furthermore, the subjective severity of sleep disturbance was linearly associated with a more significant Aβ brain burden in the APOE ε4 + group.

**Conclusion:**

This study demonstrated that CN individuals with both APOE ε4 + status and sleep disturbance exhibited greater Aβ burden. Understanding the relationship between sleep and Aβ in CN older adults may inform sleep interventions that could reduce early Aβ accumulation and delay the onset of cognitive dysfunction associated with early AD.

## Introduction

Alzheimer’s disease (AD) represents the most prevalent form of dementia among older adults. With global population aging, AD has become an increasingly critical public health challenge (Scheltens et al., 2021). Current projections indicate dementia prevalence will increase by two-thirds in low- and middle-income countries (Patterson, 2018), double throughout Europe, and triple worldwide by 2050 (Alzheimer Europe, 2019). This concerning trajectory necessitates coordinated efforts to identify and mitigate modifiable risk factors. In 2019, the World Health Organization published its inaugural guidelines addressing cognitive decline and dementia risk reduction (Organization, W.H, 2019), encompassing lifestyle interventions, strategies targeting physical health conditions, and specialized therapeutic approaches (Organization, W.H, 2021).

Identifying the underlying pathophysiological mechanisms of AD is essential for developing effective preventive strategies. Despite ongoing scientific debate, the amyloid-β (Aβ) hypothesis remains the central framework for understanding AD pathogenesis (Scheltens et al., 2016; Frisoni et al., 2022; Hardy and Higgins, 1992). Importantly, abnormal Aβ deposition begins during the preclinical stage, approximately 15–20 years before cognitive symptoms manifest (Bateman et al., 2012). This extended preclinical window provides a critical opportunity for early detection and intervention, particularly through positron emission tomography (PET) imaging, which has become a valuable tool for differential diagnosis and clinical trial enrollment (Scheltens et al., 2016).

Among the various factors influencing Aβ dynamics, sleep has emerged as a particularly significant modulator. Compelling evidence from human and animal studies reveals a bidirectional relationship between sleep and Aβ processing (Kang et al., 2009; Xie et al., 2013). Age-associated changes in sleep architecture—characterized by difficulties initiating and maintaining sleep—commonly affect older adults (Mander et al., 2017; Ohayon et al., 2004). Notably, these sleep alterations frequently precede typical AD manifestations and have been identified as important risk indicators before clinical symptom onset (Yaffe et al., 2011; Blackwell et al., 2006; Lysen et al., 2020; Nebes et al., 2009). The reproducible and quantifiable nature of these sleep patterns suggests potential utility as biomarkers for monitoring Aβ pathological progression and informing early intervention strategies (Insel et al., 2021; Winer et al., 2020; Ju et al., 2013).

The relationship between sleep disturbances and Aβ accumulation, however, remains incompletely understood. Previous investigations have demonstrated correlations between sleep disturbances and regional Aβ burden in the general population (Ettore et al., 2019; Gabelle et al., 2019; Insel et al., 2021; Spira et al., 2013; Winer et al., 2021). One study observed that elevated regional Aβ burden correlated with sleep quality impairments, but not with altered sleep duration, in cognitively intact late middle-aged adults (Sprecher et al., 2015). However, contrasting findings reported no association between Aβ-PET burden and poor sleep profiles in older adults (Gabelle et al., 2019; Du et al., 2023; Yoon et al., 2023; Spira et al., 2014). These incongruous results suggest that the association between sleep quality and Aβ deposition remains unclear. This connection is especially important because sleep problems often occur in people with mild cognitive impairment (MCI) and AD (Weldemichael and Grossberg, 2010; Peter-Derex et al., 2015; Zhang et al., 2022). The potential mechanism involves a deleterious cycle wherein Aβ accumulation disrupts neural networks essential for sleep regulation, while impaired sleep further facilitates Aβ deposition. To address these knowledge gaps, we extended our analyses to a well-characterized cohort of cognitively normal participants from the Alzheimer’s Disease Neuroimaging Initiative (ADNI).

Beyond sleep factors, genetic predisposition plays a crucial role in AD pathogenesis. The Apolipoprotein E (APOE) ε4 allele represents a well-established genetic risk factor for AD (Corder et al., 1993). Accumulating evidence indicates that the APOE ε4 allele contributes to pronounced Aβ pathology and impairs multiple aspects of normal brain function (Yamazaki et al., 2019; Blanchard et al., 2022; Ye et al., 2005). Beyond its associations with AD risk and Aβ deposition, the APOE ε4 gene also influences sleep regulation (Poirier et al., 1993; Harold et al., 2009; Thambisetty et al., 2010). Sleep disturbances may therefore exhibit differential effects across APOE variants (Hita-Yañez et al., 2012; Hwang et al., 2018), suggesting a complex interplay between genetics, sleep physiology, and Aβ accumulation. Multiple investigations have demonstrated that APOE ε4 significantly increases vulnerability to sleep disorders, including compromised sleep quality, altered sleep duration, and difficulties with sleep initiation or maintenance in cognitively normal adults (Drogos et al., 2016; Spira et al., 2017). However, research examining the interaction between APOE status, sleep quality, and Aβ accumulation remains limited. One study involving 184 cognitively normal older adults found no significant moderating effect of the APOE ε4 allele on the relationship between sleep parameters and brain Aβ burden (Brown et al., 2016). Given these conflicting findings and knowledge gaps, our investigation sought to elucidate the complex interrelationships between sleep disturbances, APOE ε4 status, and Aβ accumulation patterns in cognitively normal older adults.

## Methods

### ADNI population

Data for this study were downloaded from the ADNI database on April 21, 2022. ADNI is a longitudinal observational study of aging that enrolls participants diagnosed as CN, subjective memory concerns (SMC), MCI (both early and late stages), and AD dementia. This study focused solely on data from CN individuals aged 55–90 years, collected between March 2011 and October 2021. CN is defined as having no impairment in cognitive function, with a Clinical Dementia Rating Global Score (CDR-SB) of 0, a Mini-Mental State Examination (MMSE) score ranging from 24 to 30, and normal memory functioning assessed using the Logical Memory II subscale (Fuller et al., 2020). A full description of the inclusion/exclusion criteria for the ADNI study can be found at https://adni.loni.usc.edu.

### Measures

Participants provided demographic data upon enrollment, including age, sex, education level, and marital status. We also documented APOE ε4 genotype status, a known genetic risk factor associated with increased Aβ burden in the brain. Global cognitive function was assessed using the CDR-SB and MMSE.

The presence of sleep disturbance was determined using the Neuropsychiatric Inventory (NPI), a validated instrument covering 12 major behavioral domains with established interrater reliability and test–retest reliability (Cummings et al., 1994; Mega et al., 1996; Cummings, 1997). Component K of the NPI (NPI-K) assesses recent alterations in sleep patterns. Previous research has demonstrated associations between NPI-K scores and regional uptake of both 18F-flortaucipir and 18F-florbetapir uptake (Shokouhi, 2019). The NPI employs a structured hierarchical assessment approach, initiating with screening questions to identify symptoms within specific behavioral domains. According to previous studies (Elberse et al., 2024; Kim et al., 2023; Blackman et al., 2022), we established a specific screening protocol for sleep assessment in our study. Participants were categorized into two groups according to their sleep status. Those who reported sleep disturbance were classified into the sleep disturbance group, defined by an affirmative response to any of these key sleep questions: difficulty initiating sleep (K1), nighttime awakenings (excluding isolated bathroom visits with rapid sleep resumption; K2), or premature morning awakening relative to established sleep patterns (K6). Additionally, regarding the sleep assessment, we would like to clarify that the total severity score was calculated for NPI-K by summing up the severity ratings for all domains of sleep and nighttime behaviors. The sleep scores represent the sum of multiple items (higher scores indicate worse sleep quality).

We used the standardized uptake value ratio (SUVR) obtained using florbetapir-PET-AV45 to calculate Aβ burden. ADNI florbetapir PET scans were acquired using standardized ADNI PET protocols at the participating sites. FreeSurfer v7.1.1 delineated the regions of interest (ROIs). We used the structural MRI closest in time to each PET scan to rule out potential effects of brain atrophy associated with baseline MRI registration. Further details can be found in the ADNI_UCBERKELEY_AV45_Methods_01_14_21. The SUVR is defined as the ratio of the measured uptake in a target tissue ROI divided by the uptake in a reference ROI (Zasadny and Wahl, 1993). The choice of the reference ROI directly affects the sensitivity of SUVR quantification. The cerebellum has been widely used as a reference for florbetapir PET SUVR, especially in cross-sectional studies (Schwarz et al., 2017; Chiao et al., 2019). Here, we re-intensity-normalized the regional SUVR using a composite reference ROI of the whole cerebellum. The global 18F-Flortaucipir comprises frontal, anterior/posterior cingulate, lateral parietal, and lateral temporal regions.

At the time of data download, we identified 2,756 participants with available data from the three ADNI phases (ADNI-1, ADNI-2, and ADNI-3). Participants from all phases were eligible for inclusion, provided they met our study criteria. This cross-phase approach maximized our sample size by utilizing all available ADNI data. We applied systematic exclusion criteria as follows: 1,524 participants were excluded due to MCI or AD diagnoses. Additional exclusions comprised 8 participants with incomplete NPI-sleep questionnaires, 23 with missing APOE ε4 genotype data, 12 with incomplete neuropsychological assessments, and 168 individuals with non-zero CDR-SB scores. Subsequently, we applied Z-score standardization to the summary SUVR values (summarysuvr_wholecerebnorm, based on whole cerebellum reference region) and excluded 21 statistical outliers defined as values exceeding three standard deviations from the mean (|Z| > 3). Following these inclusion and exclusion procedures, 1,000 participants with complete summary SUVR data and regional measurements from 103 brain regions were retained for final analysis. A complete list of all 103 brain regions from FreeSurfer’s whole-brain segmentation, comprising the Desikan-Killiany cortical parcellation (68 regions) and FreeSurfer’s subcortical segmentation (35 additional structures), is provided in the Supplementary Table S7.

### Statistical analysis

Baseline demographic characteristics were compared using independent t-tests or analysis of variance (ANOVA) for continuous variables and Pearson χ^2^ tests for categorical variables, as appropriate.

Regional SUVR values were compared between participants with and without sleep disturbance using independent t-tests. For descriptive purposes, unadjusted means and standard deviations are presented for the overall sample and each sleep group. Linear regression analyses were performed for each brain region with regional SUVR values as the dependent variable and sleep disturbance status as the primary predictor. Participants without sleep disturbance served as the reference group. All regression models were adjusted for age, sex, years of education, and marital status. Results are presented as regression coefficients (β) with standard errors (SE) and corresponding *p*-values. *p*-values from regression analyses were corrected for multiple comparisons using the Benjamini-Hochberg false discovery rate (FDR) method.

To examine the moderating effect of APOE ε4 status on the relationship between sleep disturbance and regional amyloid burden, we conducted moderation analyses using linear regression framework with separate models fitted for each brain region. These models included sleep disturbance as the primary predictor, APOE ε4 carrier status as the moderator, and their interaction term (Sleep disturbance × APOE ε4). Participants carrying ≥1 copy of the APOE ε4 allele (i.e., ε2/ε4, ε3/ε4, and ε4/ε4) were classified as APOE ε4+, while all others were classified as APOE ε4−. We also conducted stratified linear regression analyses by APOE ε4 carrier status to examine the association between sleep disturbance and regional SUVR values separately in carriers and non-carriers.

In our regression models, sleep disturbance was coded as a binary variable (0 = no sleep disturbance, 1 = sleep disturbance present) and APOE ε4 status was coded as a binary variable (0 = non-carrier, 1 = carrier). This coding scheme means that individuals with sleep disturbance and APOE ε4 carriers serve as the reference conditions in our analyses. Therefore, negative beta coefficients indicate that the comparison group (no sleep disturbance or non-carriers) has lower SUVR values compared to the reference group (sleep disturbance present or carriers).

Additionally, we investigated the association between sleep scores measured by NPI-K and SUVR values in the APOE ε4 + group using linear regression analyses with the aforementioned covariates. To visualize regional differences in amyloid burden associated with sleep disturbance in APOE ε4 + individuals, we used AFNI (Analysis of Functional NeuroImages) and SUMA open-source software. Forest plots were generated using R software to visualize regression coefficients and confidence intervals across brain regions.

We first conducted exploratory analyses to identify brain regions showing significant associations at the uncorrected *p*-value level (*p* < 0.05), derived from regression models adjusted for age, sex, years of education, and marital status. To address the multiple comparisons issue, we applied Benjamini-Hochberg false discovery rate (FDR) correction to the analytical results across all 103 brain regions. FDR correction is particularly appropriate for neuroimaging studies due to the inter-correlations among brain regions.

All statistical analyses were performed using IBM SPSS Statistics (version 28.0; IBM Corp., Armonk, NY, USA), Mplus version 8.3, and R software version 4.0.3 (R Foundation for Statistical Computing). Statistical significance was set at *p* < 0.05.

## Results

The 1,000 CN participants in this study comprised 539 women (53.9%) and 461 men (46.1%) with a mean age of 71.9 (6.26) years, mean education of 16.79 (2.48) years, 71.8% married, and 30.6% APOE ε4+. Among the cohort, 866 individuals had no sleep disturbance, while 134 reported at least one sleep disturbance. No significant differences in age or APOE ε4 status were observed between participants with or without sleep disturbance. However, significant differences emerged in sex distribution, educational attainment, and marital status between groups. The mean MMSE score across all CN participants was 29.04 (1.42), with no significant differences in MMSE scores between those with and without sleep disturbance (Supplementary Table S1). Considering the potential influence of APOE ε4 on SUVR and sleep status, we utilized both APOE ε4 and sleep disturbance as stratification variables for secondary analyses (Table 1). Cognitive performance as measured by MMSE did not differ significantly among the four resulting groups.

**Table 1:** Participant demographics grouped by APOE ε4.

Variables	No sleep disturbance APOE ε4− (*n* = 601)	Sleep disturbance APOE ε4− (*n* = 93)	No sleep disturbance APOE ε4+ (*n* = 265)	Sleep disturbance APOE ε4+ (*n* = 41)	*p*
Age, years, mean (SD)	72.63 (6.27)	72.05 (5.38)	70.32 (6.29)	71.15 (6.14)	<0.001
Sex, number of males (%)	310 (51.58)	30 (32.26)	110 (41.51)	11 (26.83)	<0.001
Years of education, mean (SD)	16.95 (2.50)	16.40 (2.40)	16.72 (2.43)	15.90 (2.55)	0.017
Married states, married, n (%)	404 (67.22)	76 (81.72)	206 (77.74)	32 (78.05)	0.001
MMSE, score (SD)	29.02 (1.54)	29.13 (1.14)	29.06 (1.24)	28.95 (1.22)	0.879

APOE, apolipoprotein E; APOE ε4-, APOE ε4 negative; APOE ε4+, APOE ε4 positive; MMSE, Mini-Mental State Examination; SD, standard deviation.

### Effect of sleep disturbance on Aβ deposition

No significant difference in global SUVR was detected between individuals with and without sleep disturbance (1.10 ± 0.15 vs. 1.11 ± 0.17, *p* = 0.256). Further analysis of regional SUVR values using unadjusted independent t-tests revealed significant differences in 8 ROIs between participants with and without sleep disturbance, including the central corpus callosum, mid-anterior corpus callosum, left banks of superior temporal sulcus, left cuneus, left lingual gyrus, left pericalcarine, left superior temporal, and left precuneus (Supplementary Table S2). These regions represent functionally distinct neural networks with well-established roles in cognitive processing. The corpus callosum regions facilitate critical interhemispheric connectivity and information transfer between cerebral hemispheres. The temporal regions, specifically the banks of superior temporal sulcus and superior temporal cortex, are primarily involved in language processing and auditory function—cognitive domains that are characteristically impaired during AD progression. The visual cortex areas, including the cuneus, lingual gyrus, and pericalcarine cortex, are responsible for visual information processing, representing another functional domain known to be compromised with AD advancement. Notably, the precuneus serves as a pivotal hub within the default mode network (DMN), a brain network that is particularly vulnerable to early AD pathology. This selective pattern of regional amyloid deposition suggests that sleep disturbance may preferentially target specific neural networks that are known to be vulnerable in AD, rather than causing indiscriminate amyloid accumulation across the entire brain. Such network-specific effects support the hypothesis that sleep-related amyloid deposition follows established pathways of AD-related neurodegeneration.

However, after adjusting for sex, age, education, and marital status, only the left cuneus region remained statistically significant (β = 0.024, SE = 0.012, *p* = 0.04; Supplementary Table S2), indicating that participants with sleep disturbance showed higher Aβ deposition in the left cuneus compared to those without sleep disturbance. As a key component of the DMN that is highly active during resting state, the cuneus has been consistently implicated in AD pathogenesis, with Aβ accumulation preferentially starting in several core DMN regions. Recent studies have further identified the cuneus/precuneus as a central hub for brain functional connectivity alterations in sleep-related cognitive impairment (Mattioli et al., 2021; Horovitz et al., 2009; Lunsford-Avery et al., 2020). These findings collectively suggest that sleep disturbance may preferentially target Aβ deposition in vulnerable DMN regions such as the left cuneus, potentially representing an early marker of sleep-related neurodegeneration risk in CN older adults.

Given the established role of genetic factors in AD pathogenesis, we further examined whether individual genetic susceptibility modulates the relationship between sleep disturbance and Aβ deposition. APOE alleles represent established genetic risk factors for AD, with multiple studies demonstrating that APOE ε4 facilitates Aβ seeding and accelerates Aβ aggregation in cerebral tissues (Liu et al., 2017; Dodart et al., 2005). We found that the interactions between sleep disturbance and APOE ε4 were associated with regional SUVR. Complete results for all brain regions are presented in Supplementary Table S3, while Table 2 summarizes the significant findings with corresponding FDR-corrected *p*-values. We initially conducted exploratory analyses without FDR correction to identify potential regions of interest. The negative beta coefficients indicate lower SUVR values in individuals without sleep disturbance and APOE ε4 non-carriers compared to their respective reference groups, consistent with our hypothesis that sleep disturbance is associated with increased regional Aβ deposition.

**Table 2:** Effect of sleep disturbance and presence of the APOE ε4 on regional Aβ deposition.

Regions	Sleep disturbance				APOE ε4				Sleep disturbance * APOE ε4			
	β	*SE*	*p*	*q*	β	*SE*	*p*	*q*	β	*SE*	*p*	*q*
Anterior Corpus Callosum	−0.08	0.048	0.095	0.733	−0.092	0.041	0.026	0.191	0.081	0.034	0.018	0.169
Central Corpus Callosum	−0.061	0.046	0.183	0.733	−0.048	0.037	0.19	0.334	0.069	0.031	0.025	0.199
Left Caudate Nucleus	−0.07	0.037	0.054	0.506	−0.042	0.035	0.222	0.352	0.069	0.031	0.026	0.199
Left Cerebellar Cortex	0.017	0.007	0.019	0.217	0.019	0.006	0.002	0.029	−0.012	0.005	0.018	0.169
Left Choroid Plexus	−0.15	0.043	5.00E-04	0.012	−0.126	0.037	6.80E-04	0.012	0.12	0.032	1.50E-04	0.004
Left Hippocampus	−0.057	0.026	0.027	0.278	−0.049	0.023	0.034	0.217	0.049	0.02	0.015	0.169
Left Inferior Lateral Ventricle	−0.108	0.044	0.015	0.193	−0.095	0.038	0.013	0.131	0.087	0.034	0.01	0.129
Left Lateral Ventricle	−0.182	0.042	2.00E-05	0.002	−0.147	0.039	1.60E-04	0.007	0.141	0.034	3.00E-05	0.002
Mid-Anterior Corpus Callosum	−0.136	0.047	0.004	0.077	−0.115	0.041	0.005	0.064	0.127	0.035	0.309	0.721
Mid-Posterior Corpus Callosum	−0.077	0.051	0.132	0.733	−0.085	0.041	0.037	0.217	0.075	0.034	2.70E-04	0.006
Right Cerebellar Cortex	0.018	0.007	0.006	0.093	0.022	0.006	0.0002	0.007	−0.014	0.005	3.53E-03	0.052
Right Choroid Plexus	−0.134	0.038	4.25E-04	0.012	−0.12	0.034	3.80E-04	0.009	0.112	0.029	1.10E-04	0.004
Right Lateral Ventricle	−0.171	0.042	5.00E-05	0.003	−0.145	0.038	1.40E-04	0.007	0.136	0.033	4.00E-05	0.002
White Matter Hypointensities	−0.132	0.048	0.006	0.093	−0.143	0.041	4.60E-04	0.009	0.118	0.035	7.50E-04	0.013

APOE, apolipoprotein E; SUVR, Aβ positron emission tomographic standardized uptake value ratio. β = standardized regression coefficient; SE = standard error. Effects of sleep disturbance expressed for presence vs. absence of sleep disturbance. Effects of APOE ε4 expressed for presence vs. absence of APOE ε4 allele. Linear regression models adjusted for age, sex, years of education, and marital status. *p* values are results of linear regression analyses. *q* values are FDR-corrected p values (Benjamini-Hochberg method) for multiple comparisons across brain regions.

We reported effects of sleep disturbance and presence of the APOE ε4 on regional SUVR, presenting brain regions showing significant associations in covariate-adjusted regression analyses. Before FDR correction, 13 brain regions showed significant associations with sleep disturbance or sleep disturbance*APOE ε4 interactions after adjusting for age, sex, years of education, and marital status, including multiple corpus callosum regions (anterior, central, mid-anterior, and mid-posterior), left caudate nucleus, bilateral cerebellar cortex, bilateral choroid plexus, left hippocampus, bilateral inferior lateral and lateral ventricles, and white matter hypointensities.

Following FDR correction for multiple comparisons, several regions showed significant sleep disturbance*APOE ε4 interactions, including the left choroid plexus (β = 0.120, SE = 0.032, *p* = 1.50 × 10^−4^, *q* = 0.004), bilateral lateral ventricles (left: β = 0.141, SE = 0.034, *p* = 3.00 × 10^−5^, *q* = 0.002; right: β = 0.136, SE = 0.033, *p* = 4.00 × 10^−5^, *q* = 0.002), mid-posterior corpus callosum (β = 0.075, SE = 0.034, *p* = 2.70 × 10^−4^, *q* = 0.006), right choroid plexus (β = 0.112, SE = 0.029, *p* = 1.10 × 10^−4^, *q* = 0.004), and white matter hypointensities (β = 0.118, SE = 0.035, *p* = 7.50 × 10^−4^, *q* = 0.013). Notably, a significant sleep disturbance*APOE ε4 interaction was observed in the left hippocampus (β = 0.049, SE = 0.020, *p* = 0.015, *q* = 0.169). This finding is particularly meaningful given the hippocampus’s critical role in memory formation and its susceptibility in early AD pathogenesis.

The distribution pattern of these regions suggests that sleep disturbance may preferentially affect DMN-related structures and their supporting systems. Specifically, DMN core impairment is evidenced by Aβ deposition in the hippocampus, a key DMN node directly involved in memory processing. DMN connectivity disruption may result from corpus callosum Aβ deposition, which could compromise interhemispheric connections critical for DMN function. Additionally, the observed Aβ deposition in the ventricular system and choroid plexus suggests potential clearance system impairment, which may affect glymphatic clearance mechanisms that are crucial for Aβ removal during sleep.

### Effect of sleep disturbance on Aβ deposition grouped by APOE ε4

The significant interaction analysis results prompted us to perform subgroup analyses, and participants were divided into two groups based on APOE ε4 status (Table 3; Supplementary Tables S4, S5). Among APOE ε4 non-carriers, bilateral lateral ventricle SUVR was significantly decreased in those with sleep disturbance (left: β = −0.039, SE = 0.017, *p* = 0.019, *q* = 0.140 and right: β = −0.034, SE = 0.017, *p* = 0.042, *q* = 0.140) after adjusting for age, sex, education, and marital status (Supplementary Table S4). Although these associations did not remain significant after FDR correction, this finding was contrary to our expected results. In contrast, when analyzing the entire population without considering APOE status and focusing only on sleep disturbance (Table 2), both brain regions showed a trend toward increased SUVR. Moreover, among APOE ε4 carriers, both regions demonstrated significantly elevated SUVR that remained significant even after FDR correction (Supplementary Table S5). This pattern suggests different responses to sleep disturbance between APOE ε4 carriers and non-carriers in lateral ventricular regions.

**Table 3:** Significant regional brain Aβ deposition differences between individuals with and without sleep disturbances by APOE4 status.

	Regions	Overall	No sleep disturbance	Sleep disturbance	*p* ^a^	Linear regression				
						β	*SE*	*p* ^b^	95% CI	*q*
APOE ε4+	Mid-Posterior Corpus Callosum	1.25 (0.18)	1.24 (0.18)	1.36 (0.21)	<0.001	0.119	0.028	0.00003	[0.064, 0.174]	0.003
	Left Choroid Plexus	0.91 (0.16)	0.90 (0.15)	0.99 (0.18)	<0.001	0.094	0.025	0.00019	[0.045, 0.143]	0.005
	Right Choroid Plexus	0.93 (0.15)	0.92 (0.15)	1.01 (0.17)	<0.001	0.088	0.024	0.00025	[0.041, 0.135]	0.005
	White Matter Hypointensities	1.53 (0.17)	1.52 (0.16)	1.63 (0.19)	<0.001	0.103	0.028	0.00026	[0.048, 0.158]	0.005
	Left Lateral Ventricle	0.86 (0.18)	0.84 (0.16)	0.95 (0.22)	<0.001	0.096	0.026	0.0003	[0.045, 0.147]	0.005
	Right Lateral Ventricle	0.87 (0.18)	0.85 (0.17)	0.96 (0.20)	<0.001	0.098	0.026	0.0003	[0.047, 0.149]	0.005
	Left Pericalcarine	1.29 (0.17)	1.28 (0.15)	1.37 (0.24)	0.001	0.088	0.028	0.002	[0.033, 0.143]	0.029
	Central Corpus Callosum	1.34 (0.16)	1.33 (0.16)	1.42 (0.16)	0.002	0.077	0.026	0.004	[0.026, 0.128]	0.046
	Left Caudate Nucleus	1.11 (0.13)	1.10 (0.12)	1.17 (0.18)	0.002	0.064	0.022	0.004	[0.021, 0.107]	0.046
	Left Lingual Gyrus	1.09 (0.13)	1.08 (0.11)	1.14 (0.19)	0.003	0.057	0.021	0.006	[0.016, 0.098]	0.062
	Left Cuneus	1.15 (0.13)	1.14 (0.12)	1.20 (0.17)	0.007	0.058	0.021	0.007	[0.017, 0.099]	0.066
	Left Inferior Lateral Ventricle	1.31 (0.16)	1.30 (0.15)	1.37 (0.20)	0.006	0.067	0.026	0.009	[0.016, 0.118]	0.077
	Anterior Corpus Callosum	1.40 (0.19)	1.39 (0.18)	1.48 (0.19)	0.01	0.078	0.031	0.011	[0.017, 0.139]	0.081
	Right Caudate Nucleus	1.13 (0.14)	1.12 (0.13)	1.18 (0.18)	0.015	0.06	0.024	0.011	[0.013, 0.107]	0.297
	Posterior Corpus Callosum	1.62 (0.17)	1.61 (0.17)	1.69 (0.16)	0.004	0.072	0.028	0.012	[0.017, 0.127]	0.082
	Left Hippocampus	1.12 (0.10)	1.11 (0.09)	1.15 (0.12)	0.009	0.039	0.015	0.013	[0.010, 0.068]	0.34
	Left Cerebral White Matter	1.60 (0.15)	1.59 (0.14)	1.65 (0.18)	0.013	0.059	0.025	0.016	[0.010, 0.108]	0.179
	Left Banks of Superior Temporal Sulcus	1.35 (0.22)	1.34 (0.21)	1.43 (0.25)	0.023	0.08	0.036	0.025	[0.009, 0.151]	0.143
	Right Entorhinal Cortex	0.93 (0.09)	0.93 (0.09)	0.96 (0.12)	0.018	0.033	0.016	0.035	[0.002, 0.064]	0.181
	Right Inferior Lateral Ventricle	1.26 (0.14)	1.25 (0.14)	1.30 (0.15)	0.028	0.048	0.023	0.036	[0.003, 0.093]	0.246
	Left Entorhinal Cortex	0.93 (0.08)	0.92 (0.08)	0.95 (0.12)	0.028	0.029	0.014	0.037	[0.002, 0.056]	0.181
APOE ε4+	Right Cerebral White Matter	1.60 (0.15)	1.60 (0.15)	1.65 (0.17)	0.042	0.053	0.026	0.04	[0.002, 0.104]	0.447
	Left Superior Parietal	1.11 (0.17)	1.10 (0.16)	1.15 (0.19)	0.058	0.053	0.027	0.05	[0.000, 0.106]	0.597
	Left Lateral Ventricle	0.82 (0.16)	0.83 (0.17)	0.80 (0.13)	0.14	−0.039	0.017	0.019	[−0.072, 0.006]	0.140
	Right Lateral Ventricle	0.84 (0.16)	0.84 (0.17)	0.82 (0.14)	0.277	−0.034	0.017	0.042	[−0.067, 0.001]	0.348
	Left Entorhinal Cortex	0.93 (0.08)	0.92 (0.08)	0.95 (0.12)	0.028	0.029	0.014	0.037	[0.002, 0.056]	0.181
	Right Cerebral White Matter	1.60 (0.15)	1.60 (0.15)	1.65 (0.17)	0.042	0.053	0.026	0.04	[0.002, 0.104]	0.447
	Left Superior Parietal	1.11 (0.17)	1.10 (0.16)	1.15 (0.19)	0.058	0.053	0.027	0.05	[0.000, 0.106]	0.597
APOE ε4−	Left Lateral Ventricle	0.82 (0.16)	0.83 (0.17)	0.80 (0.13)	0.14	−0.039	0.017	0.019	[−0.072, 0.006]	0.140
	Right Lateral Ventricle	0.84 (0.16)	0.84 (0.17)	0.82 (0.14)	0.277	−0.034	0.017	0.042	[−0.067, 0.001]	0.348

APOE, apolipoprotein E. Data presented as mean (standard error). β = standardized regression coefficient; SE = standard error; CI = confidence interval. Effects of sleep disturbance expressed for presence vs. absence of sleep disturbance. *q* values are false discovery rate-corrected *p* values using the Benjamini-Hochberg method to control for multiple comparisons across brain regions within each predictor category.

a *p* values are the results of unpaired *t* tests.

b *p* values are results of linear regression analyses. Models adjusted for age, sex, years of education, and marital status.

**Figure 1:**
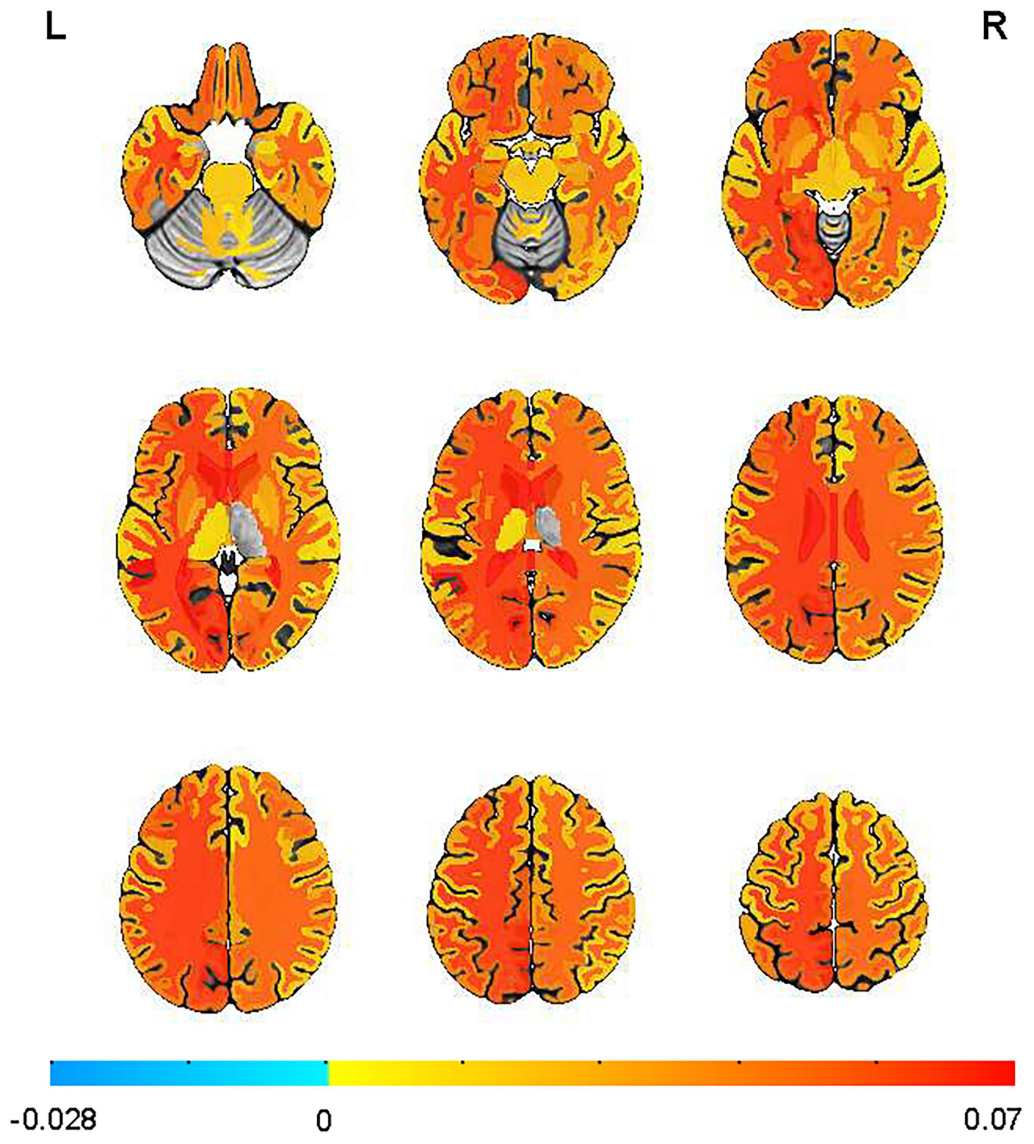
Sleep disturbances increased the mean standardized uptake value ratio (SUVR) obtained by florbetapir-PET-AV45 in the APOE ε4 + individuals. Mean images were generated by separately computing the mean of images from individuals with or without sleep disturbance. Supra-threshold clusters are presented in colors from blue to red.

Among APOE ε4 + participants, sleep disturbance was associated with significantly elevated Aβ SUVR across multiple brain regions after controlling for the same confounding variables (Figure 1). APOE ε4 carriers with sleep disturbance exhibited significantly higher Aβ deposition in multiple corpus callosum regions, including the anterior (β = 0.078, SE = 0.031, *p* = 0.011), central (β = 0.077, SE = 0.026, *p* = 0.004), mid-posterior (β = 0.119, SE = 0.028, *p* = 0.00003), and posterior (β = 0.072, SE = 0.028, *p* = 0.012) segments. Elevated SUVR values were also observed in cortical regions including the left banks of the superior temporal sulcus (β = 0.080, SE = 0.036, *p* = 0.025), left cuneus (β = 0.058, SE = 0.021, *p* = 0.007), left entorhinal cortex (β = 0.029, SE = 0.014, *p* = 0.037), left lingual gyrus (β = 0.057, SE = 0.021, *p* = 0.006), left pericalcarine cortex (β = 0.088, SE = 0.028, *p* = 0.002), left superior parietal lobule (*p* = 0.05), and right entorhinal cortex (*p* = 0.035). The left precuneus showed a trend toward significance (β = 0.068, SE = 0.035, *p* = 0.052). Notably, increased SUVR was also found in the bilateral hippocampus (left: β = 0.039, SE = 0.015, *p* = 0.013 and right: β = 0.025, SE = 0.015, *p* = 0.096).

Furthermore, sleep disturbance was significantly associated with increased SUVR in subcortical structures, including the bilateral caudate nuclei (left: β = 0.064, SE = 0.022, *p* = 0.004, *q* = 0.046 and right: β = 0.060, SE = 0.024, *p* = 0.011, *q* = 0.297), bilateral cerebral white matter (left: β = 0.059, SE = 0.025, *p* = 0.016 and right: β = 0.042, SE = 0.053, *p* = 0.026, *q* = 0.040), bilateral choroid plexus (left: β = 0.094, SE = 0.025, *p* = 0.00019, *q* = 0.005 and right: β = 0.088, SE = 0.024, *p* = 0.00025, *q* = 0.005), bilateral inferior lateral ventricles (left: β = 0.067, SE = 0.026, *p* = 0.009 and right: β = 0.048, SE = 0.023, *p* = 0.036), bilateral lateral ventricles (left: *β* = 0.096, SE = 0.026, *p* = 0.0003, *q* = 0.005 and right: β = 0.098, SE = 0.026, *p* = 0.0003, *q* = 0.005), and white matter hypointensities (β = 0.103, SE = 0.028, *p* = 0.00026, *q* = 0.005).

**Figure 2:**
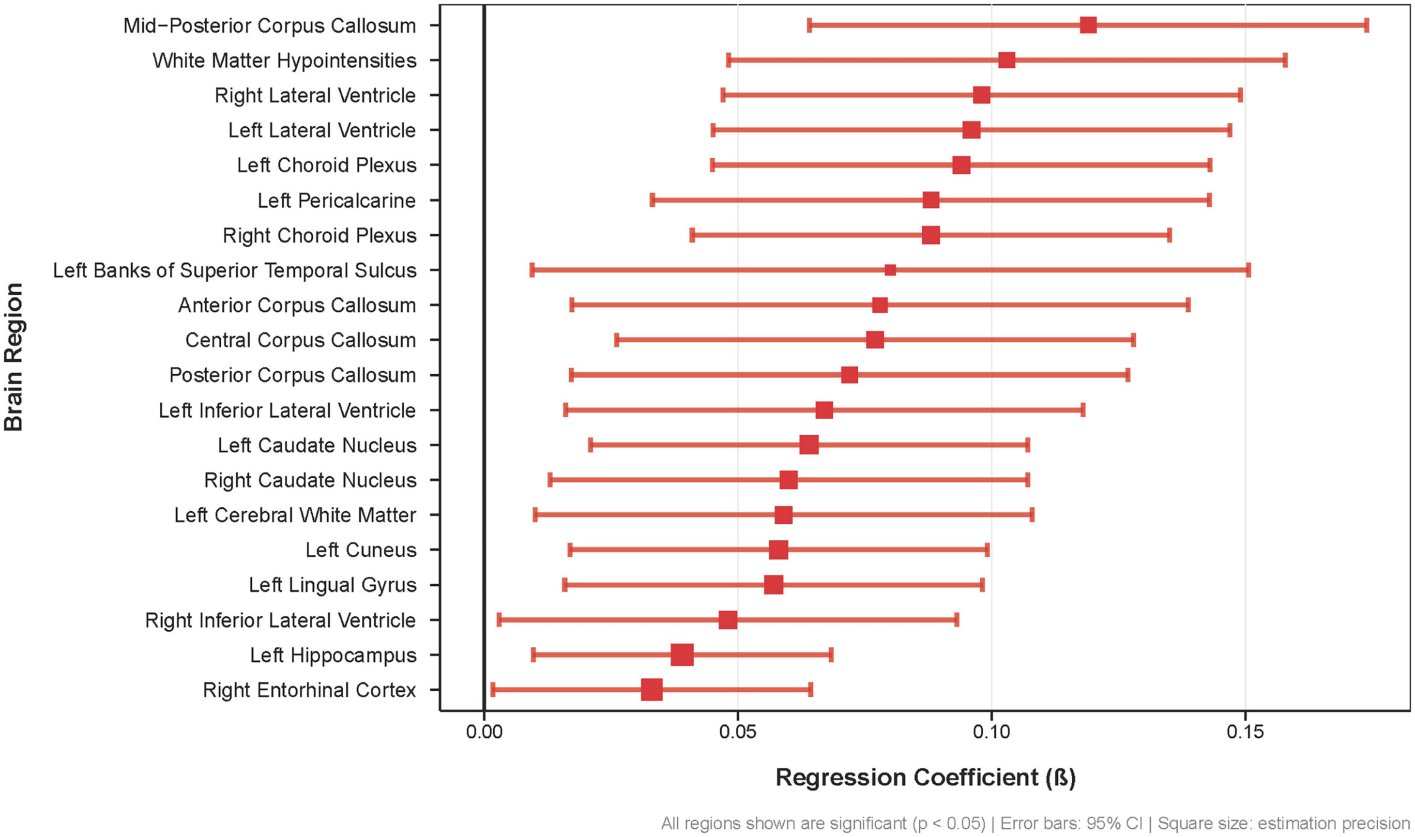
Regional brain Aß deposition differences associated with sleep disturbances in APOE £4 carriers. Forest plot showing regression coefficients (β) for the 20 brain regions with the most significant associations between sleep disturbances and Aß burden (all *p* < 0.05) Error bars represent 95% confidence intervals; square size indicates estimation precision.

Here we present the 20 brain regions demonstrating the most pronounced differences in regional amyloid-β burden between APOE4 carriers experiencing sleep disturbances and those without sleep complaints, highlighting the selective vulnerability of specific neural networks in this genetically at-risk population (Figure.2). This widespread pattern of Aβ accumulation in APOE ε4 carriers encompasses key regions of the DMN (cuneus, precuneus, entorhinal cortex and hippocampus), interhemispheric connections (corpus callosum), and cerebrospinal fluid circulation pathways (lateral ventricles, choroid plexus), indicating that sleep disturbance is associated with Aβ deposition across multiple anatomically and functionally distinct brain systems in APOE ε4 carriers.

### Effects of sleep scores on Aβ deposition in APOE ε4 carriers

We investigated whether subjective sleep disturbance severity in APOE ε4 carriers demonstrated linear associations with regional Aβ burden (Table 4; Supplementary Tables S6). All analyses were adjusted for age, sex, education, and marital status.

**Table 4:** Associations between sleep quality scores and brain Aβ deposition in APOE4 carriers.

Brain region	β (SE)	t	*p*	95% CI	q
Anterior Corpus Callosum	0.026 (0.01)	2.66	0.008	[0.007, 0.044]	0.048
Central Corpus Callosum	0.025 (0.008)	3.01	0.00	[0.009, 0.041]	1.03E-08
Mid-Anterior Corpus Callosum	0.017 (0.009)	1.97	0.05	[0, 0.033]	0.139
Mid-Posterior Corpus Callosum	0.038 (0.009)	4.33	1.49E-05	[0.021, 0.056]	0.001
Posterior Corpus Callosum	0.023 (0.009)	2.61	0.009	[0.006, 0.041]	0.052
Left Banks of Superior Temporal Sulcus	0.027 (0.011)	2.4	0.02	[0.005, 0.049]	0.077
Left Caudal Anterior Cingulate	0.017 (0.01)	1.73	0.09	[−0.002, 0.037]	0.182
Left Caudal Middle Frontal	0.017 (0.01)	1.78	0.08	[−0.002, 0.036]	0.172
Left Cuneus	0.017 (0.007)	2.55	0.01	[0.004, 0.03]	0.054
Left Entorhinal Cortex	0.014 (0.004)	3.29	0.001	[0.006, 0.023]	0.009
Left Insula	0.015 (0.007)	2.05	0.041	[0.001, 0.03]	0.124
Left Lateral Orbitofrontal	0.021 (0.009)	2.32	0.021	[0.003, 0.038]	0.077
Left Lingual Gyrus	0.016 (0.006)	2.48	0.014	[0.003, 0.029]	0.066
Left Paracentral Lobule	0.019 (0.008)	2.41	0.017	[0.004, 0.035]	0.070
Left Pericalcarine	0.026 (0.009)	2.95	0.003	[0.009, 0.043]	0.024
Left Posterior Cingulate	0.023 (0.01)	2.2	0.028	[0.003, 0.043]	0.099
Left Precuneus	0.026 (0.011)	2.44	0.015	[0.005, 0.048]	0.067
Left Superior Parietal	0.02 (0.008)	2.41	0.017	[0.004, 0.037]	0.070
Left Temporal Pole	0.014 (0.006)	2.32	0.021	[0.002, 0.026]	0.077
Left Transverse Temporal	0.01 (0.009)	1.11	0.27	[−0.007, 0.027]	0.343
Right Entorhinal Cortex	0.017 (0.005)	3.46	5.40E-04	[0.007, 0.026]	0.009
Right Lateral Orbitofrontal	0.019 (0.009)	2.03	0.044	[0.001, 0.038]	0.130
Right Paracentral Lobule	0.018 (0.008)	2.14	0.033	[0.001, 0.035]	0.106
Left Caudate Nucleus	0.023 (0.007)	3.35	0.0008	[0.01, 0.037]	0.009
Left Cerebellar Cortex	−0.003 (0.001)	−2.17	0.031	[−0.005, 0]	0.106
Left Cerebellar White Matter	0.012 (0.005)	2.5	0.013	[0.003, 0.021]	0.064
Left Cerebral White Matter	0.025 (0.008)	3.29	0.001	[0.01, 0.04]	0.009
Left Choroid Plexus	0.028 (0.008)	3.55	0.0004	[0.012, 0.043]	0.008
Left Hippocampus	0.014 (0.005)	2.83	0.005	[0.004, 0.023]	0.037
Left Inferior Lateral Ventricle	0.027 (0.008)	3.33	0.0009	[0.011, 0.042]	0.009
Left Lateral Ventricle	0.027 (0.008)	3.25	0.001	[0.011, 0.043]	0.009
Left Globus Pallidus	0.014 (0.007)	2.06	0.04	[0.001, 0.026]	0.124
Left Putamen	0.018 (0.007)	2.52	0.012	[0.004, 0.032]	0.062
Right Caudate Nucleus	0.02 (0.007)	2.68	0.008	[0.005, 0.034]	0.048
Right Cerebellar White Matter	0.01(0.005)	1.998	0.047	[2e-04, 0.0193]	0.135
Right Cerebellar Cortex	−0.002 (0.001)	−1.77	0.077	[−0.005, 0]	0.172
Right Cerebral White Matter	0.022 (0.008)	2.75	0.006	[0.006, 0.038]	0.041
Right Choroid Plexus	0.027 (0.007)	3.69	0.0002	[0.013, 0.042]	0.005
Right Hippocampus	0.009 (0.005)	1.81	0.072	[−0.001, 0.018]	0.167
Right Inferior Lateral Ventricle	0.015 (0.007)	2.14	0.033	[0.001, 0.029]	0.106
Right Lateral Ventricle	0.027 (0.008)	3.29	0.001	[0.011, 0.043]	0.009
White Matter Hypointensities	0.04 (0.009)	4.64	3.49E-06	[0.023, 0.057]	1.80E-04

APOE, apolipoprotein E. β = regression coefficient; SE = standard error; t = t-statistic; CI = confidence interval. Linear regression models examining associations between sleep quality scores and brain amyloid deposition in APOE4 gene carriers, controlling for age, education, gender, and marital status. *p* values are results of linear regression analyses for covariate-adjusted associations. *q* values are false discovery rate-corrected *p* values using the Benjamini-Hochberg method to control for multiple comparisons across brain regions within each predictor category.

**Figure 3:**
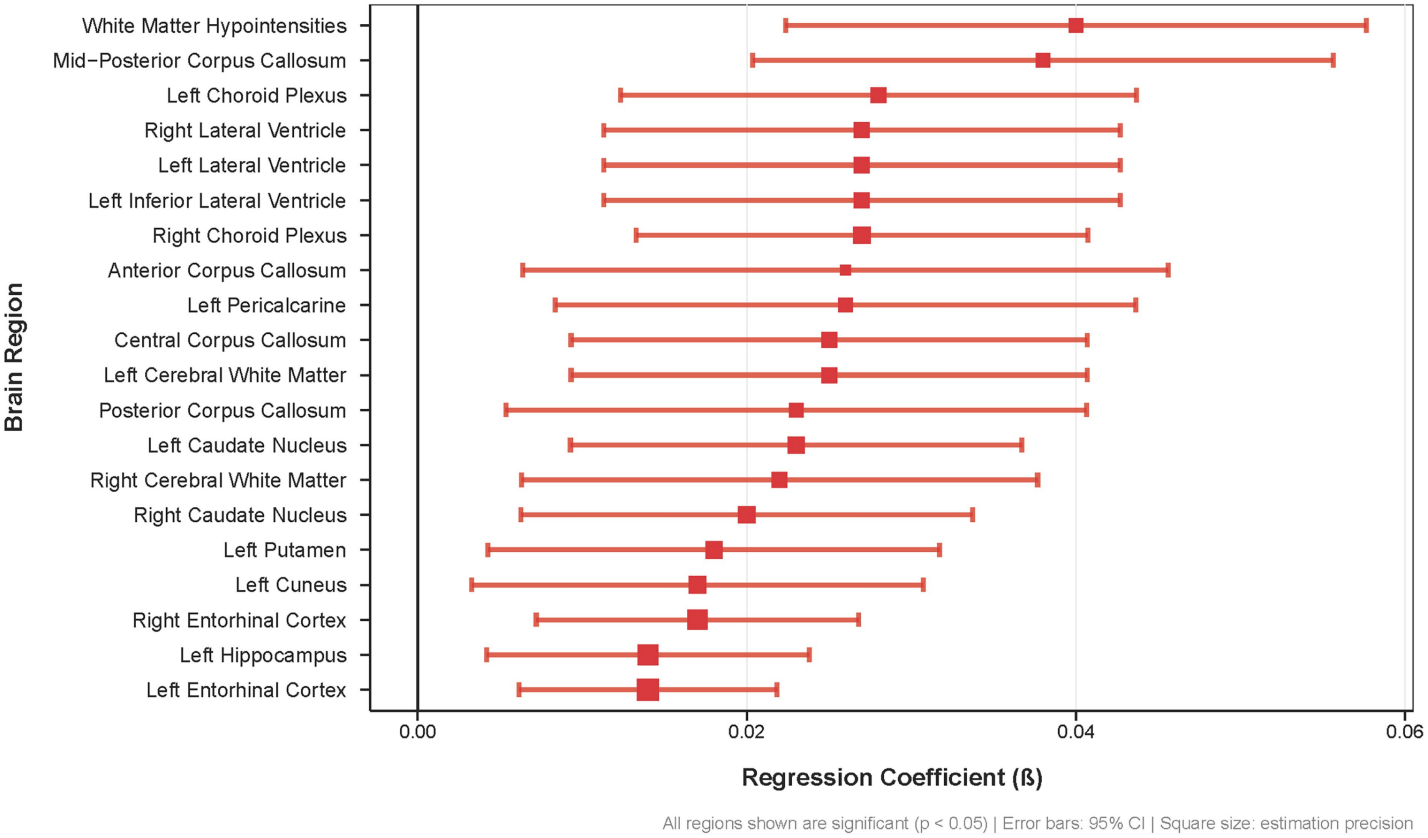
Association between sleep quality scores and regional brain Aß deposition in APOE £4 carriers. Forest plot showing regression coefficients (β) for the 20 brain regions with the strongest associations between sleep quality and Aẞ burden (all *p* < 0.05). Error bars represent 95% confidence intervals; square size indicates estimation precision.

This widespread pattern of Aβ accumulation in APOE ε4 carriers encompasses key regions of the DMN (precuneus, cuneus, entorhinal cortex, hippocampus), interhemispheric connections (corpus callosum), and cerebrospinal fluid circulation pathways (lateral ventricles, choroid plexus), indicating that poor sleep quality is linked to Aβ deposition across multiple brain regions in APOE ε4 carriers (Figure 3).

The distribution pattern of these regions suggests that poor sleep quality may preferentially affect DMN-related structures and their supporting systems. Specifically, DMN core impairment is evidenced by significant Aβ deposition in the left precuneus (β = 0.026, SE = 0.011, *p* = 0.015), a critical posterior DMN hub essential for self-referential processing and episodic memory retrieval. The left posterior cingulate cortex, another core DMN node, also demonstrated significant Aβ accumulation (β = 0.023, SE = 0.01, *p* = 0.028), suggesting potential disruption of the network’s metabolic and connectivity functions. Memory-related DMN components showed robust associations, with bilateral entorhinal cortex displaying strong significance (left: β = 0.014, SE = 0.004, *p* = 0.001, *q* = 0.009; right: β = 0.017, SE = 0.005, *p* = 5.40E-04, *q* = 0.009) and left hippocampus showing significant Aβ deposition (β = 0.014, SE = 0.005, *p* = 0.005, *q* = 0.037). Visual cortex DMN components, including the cuneus (β = 0.017, SE = 0.007, *p* = 0.01) and pericalcarine cortex (β = 0.026, SE = 0.009, *p* = 0.003, *q* = 0.024), also exhibited significant associations, indicating potential disruption of visual–spatial processing within the DMN framework. DMN connectivity disruption may result from extensive corpus callosum Aβ deposition across multiple segments (anterior: β = 0.026, SE = 0.01, *p* = 0.008; central: β = 0.025, SE = 0.008, *p* = 0.003; mid-posterior: β = 0.038, SE = 0.009, *p* = 1.49E-05), which could compromise interhemispheric connections critical for DMN synchronization and function. Additionally, the observed Aβ deposition in the bilateral lateral ventricles (left: β = 0.027, SE = 0.008, *p* = 0.001, *q* = 0.009; right: β = 0.027, SE = 0.008, *p* = 0.001, *q* = 0.009) and choroid plexus (left: β = 0.028, SE = 0.008, *p* = 0.0004, *q* = 0.008; right: β = 0.027, SE = 0.007, *p* = 0.0002, *q* = 0.005) suggests potential clearance system impairment, which may affect glymphatic clearance mechanisms that are crucial for Aβ removal during sleep.

## Discussion

This study examined the combined effects of sleep disturbance and APOE ε4 genotype on Aβ accumulation in cognitively normal older adults. Our main finding was that sleep disturbance alone did not significantly alter Aβ deposition. However, we observed a notable interaction between sleep disturbance and APOE ε4, with the left hippocampus emerging as a key region of interest. This finding is particularly significant given the hippocampus’s critical role in memory function and its known vulnerability in AD. Further analyses revealed important differences between genetic groups. APOE ε4 carriers with sleep disturbance showed significantly higher Aβ burden across multiple brain regions compared to those with normal sleep. In contrast, non-carriers showed minimal effects. Among APOE ε4 carriers, sleep disturbance severity directly correlated with regional Aβ deposition. These results suggest that genetic risk factors and sleep quality collectively influence AD-related brain pathology.

The APOE ε4 allele represents the strongest known genetic risk factor for AD, with substantial evidence connecting it to accelerated Aβ accumulation and impaired clearanc (Liu et al., 2017; Liu et al., 2013). Our results expand this knowledge by showing that APOE ε4 carriers with sleep disturbance face increased risk for Aβ accumulation. This finding is consistent with previous studies showing that APOE ε4 carriers with sleep problems have higher risk of developing AD (Burke et al., 2016) and often experience poorer sleep quality in later life (Drogos et al., 2016). Previous research using the same sleep measurement approach as our study demonstrated that individuals with different APOE ε4 status exhibited varying sleep quality patterns (Blackman et al., 2022). Our analyses revealed that sleep disturbance was linked to higher Aβ burden in AD-sensitive regions, including the bilateral entorhinal cortex and left hippocampus, particularly in APOE ε4 carriers. Additionally, sleep quality scores showed significant relationships with regional SUVR values in this genetically at-risk group, further supporting the interaction between genetic factors and sleep disturbances in AD development.

Our analysis revealed a significant interaction effect between sleep quality and APOE genetic variants within the left hippocampus region. This observation deserves attention because of the hippocampus’s central importance in memory formation and its known early involvement in AD pathological processes (Small et al., 1999; Mormino et al., 2009). Disrupted sleep patterns can negatively impact hippocampal structure (Grydeland et al., 2021), while Aβ protein accumulation may interfere with hippocampal function through effects on neural networks and cellular health (Zhang et al., 2020; Villette et al., 2010). Previous research has documented how APOE genetic variants influence the relationship between sleep difficulties and memory performance (Baril et al., 2022). Our investigation provides further evidence for a specific relationship in APOE ε4 carriers, where sleep disturbance correlates with increased Aβ deposition, particularly affecting left hippocampal structures that typically show early decline in AD development.

Our findings demonstrate that Aβ accumulation preferentially occurs in core regions of the default mode network, including the precuneus, cuneus, hippocampus, entorhinal cortex, and posterior cingulate cortex, particularly in APOE ε4 carriers with sleep disturbance. This pattern is consistent with established knowledge of DMN vulnerability in AD and suggests that sleep-related pathological processes may accelerate within this critical network. The DMN’s high metabolic activity and extensive connectivity may render it particularly susceptible to sleep-dependent clearance impairments, especially in the context of genetic risk.

Beyond DMN core regions, we identified significant findings in visual cortex areas including the cuneus, lingual gyrus, and pericalcarine cortex, all showing increased Aβ deposition associated with sleep disturbance in APOE ε4 carriers. This pattern is consistent with brain networks showing altered functional connectivity in AD identified in a previous meta-analysis (Chiari-Correia et al., 2023). Additionally, extensive corpus callosum involvement across multiple segments suggests disruption of interhemispheric connections critical for DMN synchronization and function. The widespread pattern of Aβ deposition in corpus callosum regions indicates that sleep disturbance may compromise the structural connectivity that underlies DMN integrity in genetically susceptible individuals.

A particularly finding was the significant Aβ deposition in bilateral lateral ventricles and choroid plexus in individuals with sleep disturbance. These structures play crucial roles in cerebrospinal fluid circulation and metabolic waste clearance processes (Yan et al., 2025; Čarna et al., 2023; Schubert et al., 2019). Growing evidence suggests that sleep facilitates Aβ clearance through cerebrospinal fluid flow and glymphatic circulation (Chong et al., 2022). Our findings may provide further evidence supporting the critical role of sleep in Aβ clearance through cerebrospinal fluid metabolic circulation, suggesting that sleep disturbance may impair these clearance systems specifically in APOE ε4 carriers.

A particularly notable finding was the significant Aβ deposition in bilateral lateral ventricles and choroid plexus in individuals with sleep disturbance. These structures play crucial roles in cerebrospinal fluid circulation and metabolic waste clearance processes, providing strong evidence for the mechanistic link between sleep and Aβ clearance. Growing evidence suggests that sleep facilitates Aβ clearance through cerebrospinal fluid flow and glymphatic circulation (Achariyar et al., 2016; Serrano-Pozo et al.,2021),and our findings may provide further support for this critical relationship.

Our subgroup analyses revealed contrasting patterns between APOE ε4 carriers and non-carriers in lateral ventricular responses to sleep disturbance, providing critical insights into genotype-specific clearance mechanisms. In APOE ε4 non-carriers, the observed reduction in lateral ventricular SUVR during sleep disturbance may reflect the characteristics of cognitively normal individuals in this population, where those with higher baseline Aβ burden may have already progressed beyond the cognitively normal stage and were excluded from the study. Consequently, the remaining cognitively normal non-carriers may have relatively low baseline SUVR values with limited capacity for detectable increases, resulting in apparent decreases that likely represent measurement variability rather than meaningful biological change. This interpretation is supported by the loss of significance after FDR correction.

In contrast, APOE ε4 carriers demonstrated significantly elevated SUVR in both lateral ventricles during sleep disturbance, with associations that remained significant even after FDR correction, indicating genuine pathological changes in the clearance system. Despite having higher baseline Aβ burden, APOE ε4 carriers demonstrate continued vulnerability to sleep-related Aβ accumulation in the ventricular system, suggesting that this region retains capacity for further pathological changes even in the presence of existing amyloid pathology. Additionally, previous research has shown that cognitively normal APOE ε4 carriers and non-carriers exhibit different associations between neurodegeneration and choroid plexus volume and calcification (Ozsahin et al., 2025). This genotype-specific difference highlights that APOE ε4 carriers are more vulnerable to sleep-related pathological changes.

The bidirectional relationship between sleep regulation and Aβ metabolism provides a mechanistic framework for interpreting our results. Even short-term sleep loss significantly increases Aβ deposition in the hippocampus (Shokri-Kojori et al., 2018). Clinical studies have shown links between both very short or long sleep duration and increased Aβ burden in cognitively normal individuals (Insel et al., 2021; Spira et al., 2013; Ma et al., 2020). Poor sleep quality consistently relates to higher Aβ deposition in older adults (Ju et al., 2013; Ettore et al., 2019; Branger et al., 2016), and initial sleep quality predicts later cortical Aβ accumulation in healthy aging (Winer et al., 2020). These findings suggest that investigating the association between sleep and longitudinal changes represents an important next step.

Our results demonstrated an asymmetric distribution of sleep-related Aβ deposition, with predominantly left-sided regional associations. This pattern aligns with established observations of hemispheric asymmetry in early AD pathology, where Aβ plaques tend to deposit preferentially in the left hemisphere during preclinical stages (Yoon et al., 2021; Yu et al., 2024). Previous studies have documented left-lateralized glucose metabolism declines in amyloid-β positive individuals with mild cognitive impairment (Weise et al., 2018). Our findings suggest that sleep-related amyloid accumulation may preferentially affect the left hemisphere during preclinical stages of the disease.

The choice of tracer and reference region significantly influences SUVR quantification (Ottoy et al., 2017; Heeman et al., 2020; Chen et al., 2021). Florbetapir 18F-based PET tracers, compared with 11C-PiB, have a longer half-life and could be more widely available, readily standardized, and rapidly acquired (Brendel et al., 2017; Barthel and Sabri, 2011). Human PET studies have indicated florbetapir-PET-AV45 as an adjunct for clinical diagnosis (Johnson et al., 2013; Camus et al., 2012). In this study, we used the florbetapir-PET-AV45 SUVR referenced by the whole cerebellum to calculate the Aβ burden. A higher Aβ burden measured using florbetapir-PET-AV45 was associated with fewer hours of nightly sleep in another CN cohort population (Winer et al., 2021). While PET molecular imaging offers non-invasive assessment suitable for population-level screening, particularly in older adults, it does present limitations including relatively lower spatial resolution and considerable operational costs. These limitations highlight the value of our findings regarding sleep disturbance assessment, which could complement imaging approaches by providing an accessible clinical marker that may help identify individuals at increased risk for AD-related pathology, particularly among APOE ε4 carriers.

## Limitations

This study has several important limitations that warrant consideration. First, despite our thoroughly characterized cohort, the limited number of APOE ε4 carriers experiencing sleep disturbances constrained our ability to fully elucidate the impact of this allele on Aβ pathology. This sample size limitation particularly affects our subgroup analyses and may influence the generalizability of our findings. Additionally, our APOE genotyping data comes from the ADNI database, which provides APOE ε4 carrier status coded as the number of ε4 alleles (0, 1, or 2) rather than complete genotyping information. This coding system does not distinguish between ε3/ε4 and ε2/ε4 heterozygotes in the single ε4 allele group, which represents a significant limitation of our study design. The inclusion of ε2/ε4 heterozygotes in our ε4 carrier group may attenuate the pathological effects of ε4 due to the protective effects of ε2 on amyloid pathology (Corder et al., 1993), and brain white matter structure (Heise et al., 2024), potentially reducing our statistical power to detect ε4-related associations. Future studies with complete APOE genotyping would provide more precise estimates of ε4-specific effects on the sleep-amyloid relationship. Second, our cross-sectional design prevents establishing causal relationships between sleep disturbance and Aβ deposition. The observed associations could reflect either sleep disturbances contributing to Aβ accumulation or early, subclinical Aβ pathology disrupting sleep regulation networks. Longitudinal studies are essential to determine the temporal sequence and causal relationships between these phenomena. Third, our assessment methodology relied on sleep data derived from NPI questionnaires rather than objective measurements from actigraphy or polysomnography. Although the NPI-K has been validated as a reliable clinical assessment tool in individuals both with and without AD, it remains a subjective measure dependent on caregiver reports with potential for reporting bias. While restricting our analysis to the basic presence of sleep disturbance rather than its detailed characteristics reduced the likelihood of missing clinically significant sleep abnormalities, this methodological approach lacks the precision offered by laboratory-based sleep monitoring. Finally, our analytical approach did not distinguish among various types of sleep disturbances, which may impact Aβ accumulation differently. Future investigations should characterize how specific sleep parameters (such as sleep duration, efficiency, or architecture) influence Aβ deposition patterns to better understand their relationship with AD risk.

## Conclusion

This study examined the complex interplay between sleep disturbance, APOE ε4 status, and Aβ burden in cognitively normal older adults. Our initial analyses revealed no significant differences in global PET SUVR between individuals with and without sleep disturbances, nor in regional SUVR after adjusting for demographic variables. However, subsequent analyses demonstrated significant interaction effects between sleep disturbance and APOE ε4 genotype. In stratified analyses, APOE ε4 carriers with sleep disturbance exhibited significantly elevated Aβ burden across multiple AD-vulnerable regions, particularly in the entorhinal cortex, left hippocampus, and key components of the default mode network. Moreover, among APOE ε4 carriers, sleep disturbance severity demonstrated dose-dependent associations with regional Aβ deposition. These findings support the hypothesis that sleep quality influences Aβ deposition and neurodegenerative processes, with genetic vulnerability modulating this relationship (Wang and Holtzman, 2020). Future longitudinal investigations are warranted to elucidate the temporal and mechanistic relationships between sleep quality, APOE genotype, and Aβ accumulation. Such research may provide valuable insights for early AD detection and prevention strategies. By identifying the interactions between sleep disturbance, APOE ε4 status, and Aβ burden, this study contributes to the development of risk stratification approaches and potential intervention targets for individuals at increased risk for AD.

## Data Availability

The original contributions presented in the study are included in the article/Supplementary material, further inquiries can be directed to the corresponding authors.
